# Applicability Assessment of Different Materials for Standards Ensuring Comparability of Optical and Tactile Coordinate Measurements

**DOI:** 10.3390/ma15124128

**Published:** 2022-06-10

**Authors:** Wiktor Harmatys, Piotr Gąska, Adam Gąska, Maciej Gruza, Michał Jedynak, Konrad Kobiela, Michael Marxer

**Affiliations:** 1 Laboratory of Coordinate Metrology, Cracow University of Technology, al. Jana Pawla II 37, 31-864 Krakow, Poland; wharmatys@pk.edu.pl (W.H.); maciej.gruza@pk.edu.pl (M.G.); michal.jedynak@pk.edu.pl (M.J.); konrad.kobiela@pk.edu.pl (K.K.); 2 Department of Manufacturing Systems, Faculty of Mechanical Engineering and Robotics, AGH University of Science and Technology, al. Mickiewicza 30, 30-059 Krakow, Poland; gaska@agh.edu.pl; 3 Institute for Production Metrology, Materials and Optics, Eastern Switzerland University of Applied Sciences, Werdenbergstrasse 4, 9471 Buchs, SG, Switzerland; michael.marxer@ost.ch

**Keywords:** coordinate measuring machine, optical measurements, accuracy, multisensor measurement, video probe, white light sensor

## Abstract

Multisensor CMMs are systems with an established position on the market, but their popularity still grows, as they provide access to the advantages offered by tactile and contactless measurement methods. Yet there are still questions of the comparability of results obtained using the optical and tactile operation modes of multisensor system. This phenomenon can be assessed by measuring appropriate gauges, most often reference rings or spheres. Due to the completely different nature of probing processes for tactile and contactless measurements, the material from which reference object is made may significantly affect measurement results. In order to assess the influence of this factor on measurement accuracy, three reference spheres made from different materials were measured on optical multisensor CMMs. Measurements involved tactile measurements as well as optical measurements made using different probing systems: a video probe and white light sensor. Results obtained from performed experiments show large differences depending on the material used for spherical standard production. On the basis of obtained results, it can be stated that the best material for a reference object that can be used for comparability tests of tactile and optical measurements is a composite of alumina with at least one oxidic additive.

## 1. Introduction

Changes in coordinate measuring techniques align with requirements formulated by the fourth industrial revolution. Among them, such trends can be pointed out as: acceleration of measurement process, integration of different production process stages and enlargement of capabilities of measuring devices as well as enhancement of range of their possible functionalities. The last of the abovementioned trends can be observed in the development of so-called multisensor CMMs, which combine the advantages of tactile and contactless measuring systems. Their working principle and the main components that are used during measurement are described in [1,2,3]. Examples of application of mulisensor CMMs in industrial practice are given in [4,5,6], while the directions for the future development of systems of such a type, with special emphasis put on accuracy improvement, are described in [7,8,9]. In systems of such a type, tactile measurements can be applied for datum definition or for measurements of dimensions with narrow tolerance zones, while optical methods are utilized for controlling small dimensions and to accelerate measurement processes. As multisensor CMMs use different types of probing systems, their accuracy may differ depending on the measurement task and various measurement conditions. The kinematic structure of multisensor machines is adapted from typical tactile CMMs and their errors can be described in the same manner as in case of standard CMMs [10,11,12]. Similarly, the performance and feasibilities of tactile sensors used in machines of this type are well-described and have been studied thoroughly [10,13,14]. An aspect of multisensor CMMs that has not been studied to such an extent is the accuracy of optical sensors and the influence of various factors on their behaviour. Skibicki et al. describe, in [15], how the measurement uncertainty of a vision system can be affected by factors such as the sensitivity of the image sensor, the focal length of the lens or the brightness level of recorded images. The results of their studies showed that the uncertainty of measurements performed with a vison system can change significantly depending both on the measuring-system configuration and measurements conditions. Bernstein and Weckenmann [16] discussed the influence of factors such as applied illumination, air contamination or vibrations on the measurement uncertainty of a system equipped with optical probe. Carmignato et al. [17] described experiments that involve measurements of different reference objects performed on optical CMMs, which show that factors such as illumination, auto-focus, magnification of objective or utilized measuring window size have a significant influence on the uncertainty of measurements. The performance verification of optical CMMs that are used in industry was described in [18]. Presented tests included measurements of different reference objects made of various materials. The research showed the necessity of the constant monitoring of optical CMMs and their interim verification. Another important issue related with the usage of multisensor CMMs is the consistency of results that can be obtained for the same measurement task performed in a tactile and contactless manner. This phenomenon can be assessed by conducting measurements of the same artefact using both modes of machine operation. Thus, the problem arises of the appropriate selection of material from which reference object is made. The artefacts used in coordinate metrology were reviewed in [19]. Authors indicated that the selection of the material from which the artefact is manufactured is of key importance for the results obtained during its measurement. In [20], the influence of the material used for artifact on measurement accuracy was investigated. A number of materials were tested, including: silicon nitride, quartz glass, sapphire rough and sapphire fine. Measurements of reference objects were performed tactilely and with different optical probes. Presented results indicated the small impact the of used material on measurement results and the significant influence of this factor on measurement uncertainty for video probes. The subject of the influence of the material of the measured object on measurement accuracy were also studied separately for tactile and contactless measurements. In [21], Wozniak and Dobosz assess the influence of the different qualities of the measured object on probing system pretravel. One of the tested object properties was object stiffness, expressed by Young modulus. Experiments included measurements of samples made of teflon, aluminum, silicon and ceramics. Results showed the significant influence of material stiffness on two-dimensional probe pretravel variation. Carmignato and Savio described, in [22], experiments that led to performance verification of CMMs equipped with optical systems. Presented considerations include the selection of material used for reference object manufacturing. Several different materials were studied, which resulted in the development of reference objects made from acid-etched stainless steel and sandblasted alumina. Such materials were chosen for the verification of laser triangulation probes, as their surfaces can be characterized by almost Lambertian behaviour. In [23], the influence of the surface coating of reference balls were studied. Different types of coating were used, such as: B-MOS, chromeplating, sandblasting with TiN-coating. The highest measurement repeatability was obtained in the case of an artefact with a sandblasted surface, which brought authors to the conclusion that they are adequate for test artefacts.

A comparison of the performance of measurements obtained using optical and tactile CMMs was presented in [24]. The authors describe measurements that were made on a specially developed hole plate artefact by a number of institutions, which guided authors to the conclusion that optical probes can give results comparable to those obtained with tactile CMM, but in specific situations the difference may rise significantly. Nevertheless, the determination of the coincidence error (which gives information about the consistency of results obtained for tactile and contactless measurements) is still a current problem, which is worth further investigation.

The main aim of this paper is to investigate which of the materials that are used for the manufacturing of material standards utilized in coordinate metrology provides the best comparability of optical and tactile coordinate measurements. Research presented in this paper is based on experiments consisting of measurements of spherical standards made of different materials available on market, using different measuring machines and different sensors (both tactile and optical). The novelty of the paper lies in the applied probing strategy. The points measured using different systems are the same for all sensors (their number is not the same but even when they are measured in a smaller number of points, the locations of the chosen points are the same as in the case of tactile measurements, which may be treated in this case as a reference). Another fact is that the research includes the testing of the measuring capabilities of new materials that were used specifically for the manufacturing of spherical standards for multisensor coordinate measurements. These kinds of materials are evaluated (by accredited calibration laboratories) for the first time in the state-of-the-art scholarship.

The following part of this paper presents experiments that were undertaken in order to find appropriate material for reference object that can be used for verification tests or for measurement uncertainty analysis (i.e., using the calibrated workpiece method described in [25]) for both operation modes of multisensor machines. Section 2 describes three different reference objects that were included in experiments, which are characterized by different qualities. This section also comprises specification of measuring devices which were used during tests, as well as a description of the experimental procedure, which involved the determination of reference object form error and diameter measurements. Section 3 presents the results of the performed experiments, while Section 4 includes discussion of the experiments results and presents directions of future works.

## 2. Materials and Methods

Saphirwerk calibration spheres were used in the described tests. Three reference balls, with a nominal diameter of 10 mm, were made of different materials. The first reference object was ceramic, made from alumina. This is the material most commonly used for manufacturing of calibration spheres for contact measurements. It is characterized by high hardness and good insulating properties, so it remains dimensionally stable under temperature changes. Additionally, it allows high-precision processing, which results in a high-gloss surface. The next two types of material are basically used for optical measurements. Saphirwerk has developed technology for producing high-precision spheres characterized by a matte and smooth surface. It is achieved by mixing the base substance, which is Al_2_O_3_, with at least one oxidic additive. Two different versions of the TOPIC material have been optimised to meet the requirements characteristic for different optical measurement methods. TOPIC white is recommended for optical measurements, while TOPIC black is recommended for optical-contact measurements. The characteristics of the reference spheres used in experiments are included in the Table 1.

**Figure 1:**
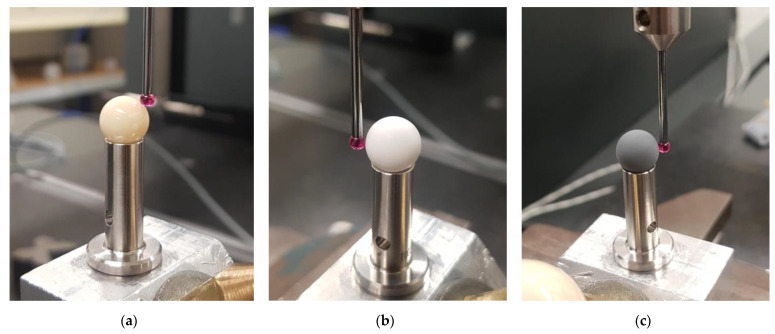
The reference objects that were utilized during experiments: (**a**) Alumina; (**b**) Topic White; (**c**) Topic Black.

In order to assess how the material of each standard influences the measurement results, all of them were measured with tactile and optical sensors, on different measuring systems. The reference objects that were utilized during experiments are presented in Figure 1.

The experiments were performed using four different machines. Two of them were located in Laboratory of Coordinate Metrology at Cracow University of Technology (Leitz PMM and Zeiss O-Inspect) and two in Institute for Production Metrology, Materials and Optics at Eastern Switzerland University of Applied Sciences in Buchs (Werth VideoCheck HA and Leitz Reference). Specifications of the coordinate-measuring machines are listed in Table 2.

**Figure 2:**
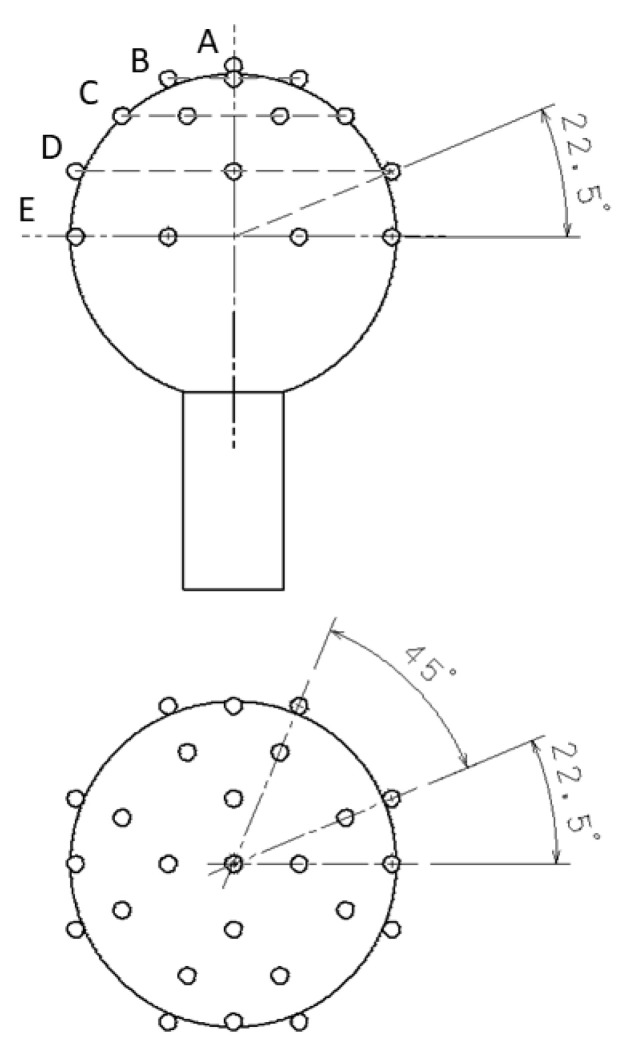
The probing strategy used during experiments for measurements of all reference balls. (**Upper**) sketch presents front view, (**lower**) sketch presents top view.

According to VDI/VDE 2617, each sphere was measured with 25 probing points. Each time, the point-by-point measurement mode was utilized and the following point pattern was proposed: 1-4-8-4-8 (A-B-C-D-E), where first point was probed at the pole of the sphere and last eight points were probed at the sphere’s equator. Levels were placed equally every 22.5 degree (elevation angle). In all tactile measurements, the 4 mm tip ball diameter was used, and the stylus length equalled 38 mm. Comparison between different measuring heads was performed with use of specific points. All points were used to compare tactile heads. For the optical sensors, 8 points at the equator (level E) were used to calculate reference circles. Measurements with probe were performed with maximum possible magnifications of utilized probing systems. Points from levels A, B and C were used to calculate reference spheres to compare WLS sensor with the reference measurement. The strategy used during measurements is presented in Figure 2. Before measurements, standard spheres were calibrated in accredited calibration laboratory with uncertainty-of-diameter calibration at the level of 0.3 µm and uncertainty-of-form-deviation calibration at the level of 0.04 µm.

All measurements were performed in accredited calibration laboratories in air-conditioned rooms with good thermal stabilities (no worse than 20 ± 0.5 °C). Thermal influences on measuring spheres is negligible due to two reasons:

(1) The biggest CTE for materials of which the spheres were manufactured is 5 × 10^−6^ K^−1^. Maximum temperature changes that may happen in laboratories with thermal stability described above are 1 °C. Diameter of the spheres is 10 mm. For this input data, maximum temperature influence on sphere diameter can be given by Equation (1):

0.000005 × 1 × 0.01 m = 0.05 µm(1)

(2) Differences in CTE of different spheres is 0.4 × 10^−6^ K^−1^, so the temperature influences them in similar way. All spheres were measured one by one with very short breaks between different spheres. In that short time, changes in temperature values were slight and all spheres were measured in comparable thermal conditions.

Next section presents results of performed measurements.

## 3. Results

**Figure 3:**
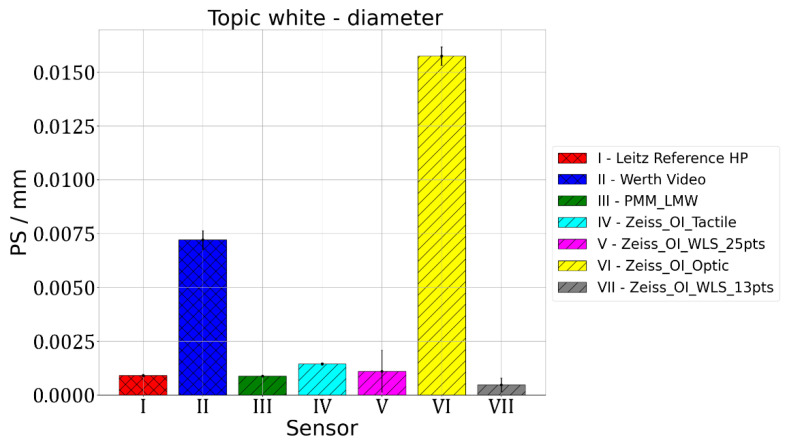
Results of measurements of sphere diameters for sphere made of Topic White.

**Figure 4:**
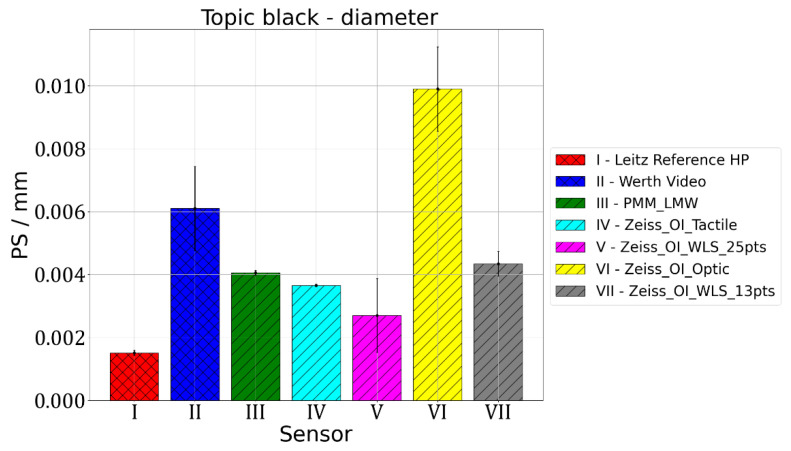
Results of measurements of sphere diameters for sphere made of Topic Black.

**Figure 5:**
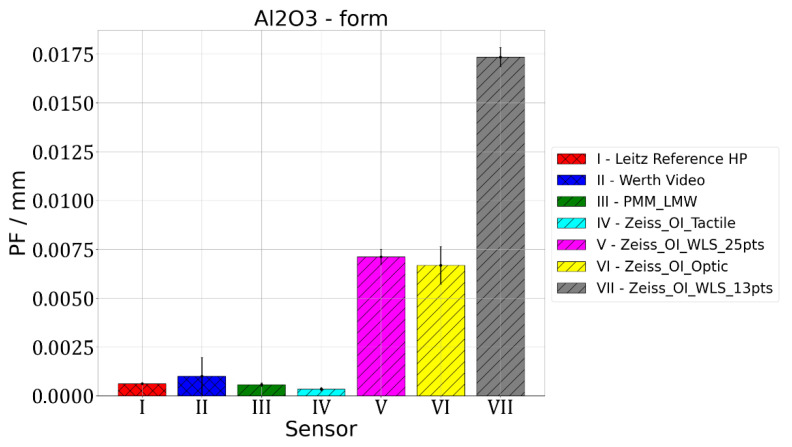
Results of measurements of PF parameter value for sphere made of alumina.

**Figure 6:**
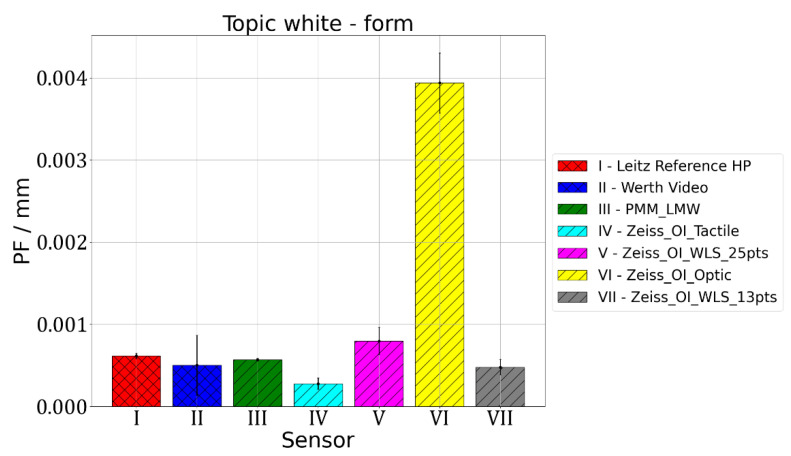
Results of measurements of PF parameter value for sphere made of Topic White.

**Figure 7:**
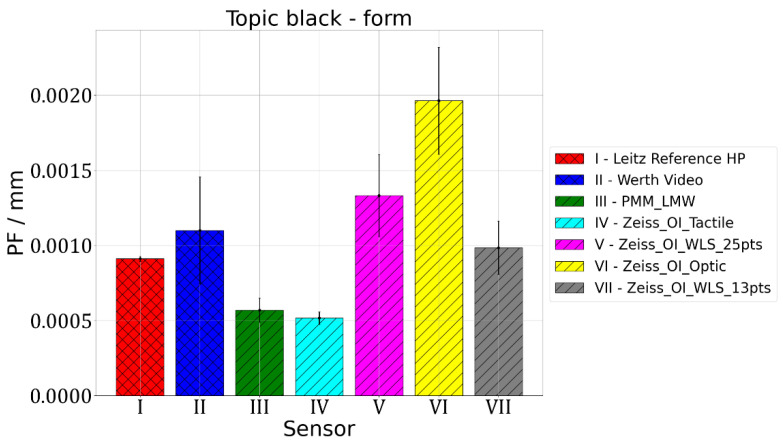
Results of measurements of PF parameter value for sphere made of Topic Black.

For the measurement of standard spheres, three parameters are usually assessed. The first one is used to check how accurately the diameter of the sphere was determined. According to VDI/VDE 2617, this parameter is called PS and is calculated as a difference between diameter measured on the considered CMM and the reference value taken from the calibration certificate of the standard sphere. The results of the measurements of spheres diameters are presented in Figure 3 and Figure 4. The PS parameter gives information about the deviation between the measurement result and the diameter of the reference ball given in calibration certificate. The following abbreviations were used in Figure 3, Figure 4, Figure 5, Figure 6 and Figure 7: Leitz Reference HP—measurements performed using the Leitz Reference HP machine equipped with tactile measuring head, Werth Video—measurements performed using Multisensor Werth VideoCheck HA machine equipped with video probe, PMM_LMW—measurements performed using Leitz PMM 12106 machine equipped with tactile measuring head, Zeiss_OI_Tactile—measurements performed using Multisensor Zeiss O-Inspect 442 machine equipped with tactile measuring head, Zeiss_OI_WLS_25pts—measurements performed using Multisensor Zeiss O-Inspect 442 machine equipped with chromatic white light sensor using 25 measuring points, Zeiss_OI_Optic—measurements performed using Multisensor Zeiss O-Inspect 442 machine equipped with video probe, Zeiss_OI_WLS 13pts—measurements performed using Multisensor Zeiss O-Inspect 442 machine equipped with chromatic white light sensor using 13 measuring points.

For Leitz Reference, the MPE for size measurements equalled 0.5 µm and the maximum observed PS value of 1.5 µm was obtained for the Topic Black sphere. For Wert VideoCheck, the MPE for size measurements equalled 1.5 µm and the maximum observed PS value of 7.2 µm was obtained for the Topic White sphere. For Leitz PMM, the MPE for size measurements equalled 0.8 µm and the maximum observed PS value of 4.1 µm was obtained for the Topic Black sphere. For Zeiss O-Inspect, the biggest value of PS, which equalled 15.8 µm, was obtained for the Topic White sphere when it was measured using the Video probe, for which MPE for size measurements was 1.9 µm.

The second parameter that was assessed is PF parameter, which is related to the accuracy of form-deviation measurement. All the measured points are used to calculate the best-fit Gaussian sphere, then radial distances from the centre of the fitted sphere to each measuring point are determined and the difference between the maximum and minimum radii obtained this way is the value of the PF parameter. The results of the measurements of the PF parameter value are given in Figure 5, Figure 6 and Figure 7.

For Leitz Reference, the MPE for form measurements equalled 0.5 µm and the maximum observed PF value of 0.9 µm was obtained for the Topic Black sphere. For Wert VideoCheck, the MPE for form measurements equalled 1.3 µm and the maximum observed PF of 1.1 µm was obtained for the Topic Black sphere. For Leitz PMM, the MPE for form measurements equalled 0.6 µm and for all the measured spheres, a similar value of PF, which equalled about 0.6 µm, was obtained. For Zeiss O-Inspect, the biggest value of PF, which equalled 17.4 µm, was obtained for the Alumina sphere when it was measured using a chromatic white light sensor in 13 points. For this probing system, the MPE for form measurements is 2 µm.

The third parameter that was assessed was related to the location of sphere centre. During the measurement of sphere each time, the origin of the part coordinate system was set in the centre of the sphere measured manually and, after that, recreated automatically (in order to get rid of error related to manual measurements). The measurements of spheres using the strategy presented in the previous section were performed after the definition of the part coordinate system. The results of the determination of sphere centres are presented in Table 3.

**Figure 8:**
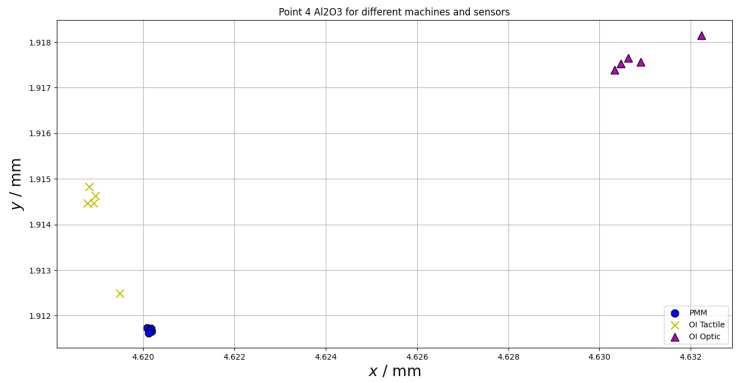
Repeatability of measuring point for chosen point at the equator of the sphere made of alumina.

**Figure 9:**
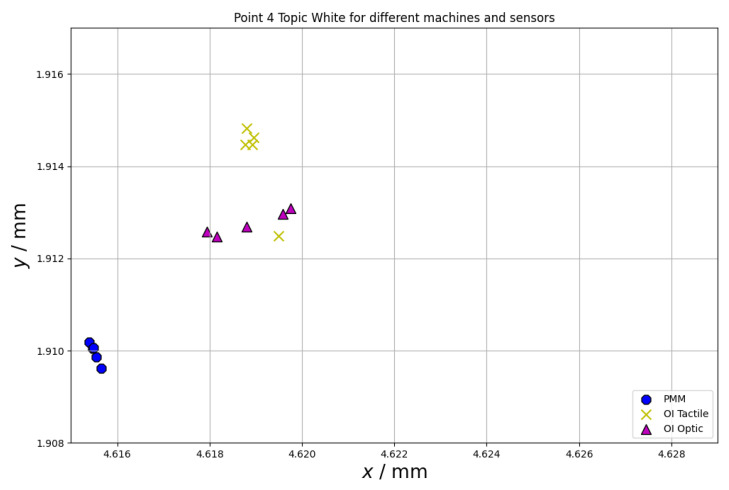
Repeatability of measuring point for chosen point at the equator of the sphere made of Topic White.

**Figure 10:**
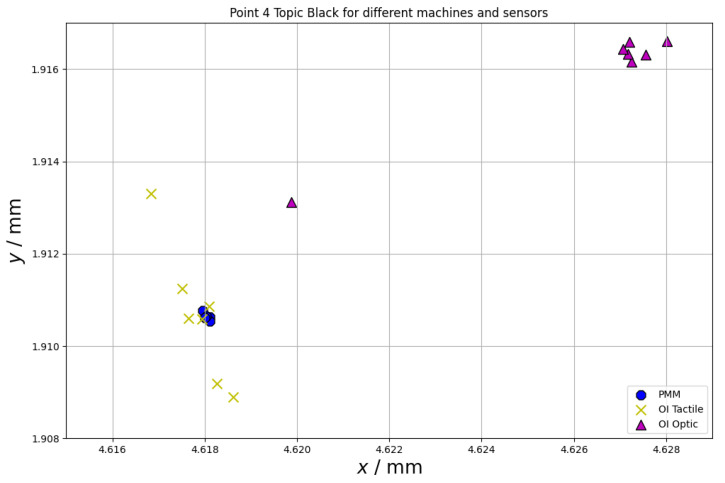
Repeatability of measuring point for chosen point at the equator of the sphere made of Topic Black.

Another important error source during measurements on CMMs using different probe types is the repeatability of the measuring point. Both for the tactile and optical measurements, this may be affected by material properties (surface parameters, such as surface roughness, for both tactile and optical measurements and optical parameters, such as refractive index or reflectivity, for optical measurements). The repeatability of the measuring point was also investigated within the scope of this paper. The results of the measurements of the chosen point at the equator of the sphere (for all spheres the same point was considered) are presented in Figure 8, Figure 9 and Figure 10. The following abbreviations were used in Figure 8, Figure 9 and Figure 10: PMM—measurements performed using Leitz PMM 12106 machine equipped with tactile measuring head, OI Tactile—measurements performed using Multisensor Zeiss O-Inspect 442 machine equipped with tactile measuring head, OI Optic—measurements performed using Multisensor Zeiss O-Inspect 442 machine equipped with video probe.

Similar results were also obtained for points measured from different directions.

## 4. Discussion

The measurements of standard spheres’ diameter and the determination of PS parameter values show differences between the different materials and different probe heads used. For Topic White, the mean PS error calculated for all measurements performed on all machines and using all sensors equalled 0.0039 mm and the range of PS parameter values was at the level of 0.0154 mm, with the minimum error for measurements using the white light sensor (0.0004 mm) and maximum error for the O-Inspect machine equipped with video probe (0.0154 mm). For Topic Black, the mean PS error was 0.0046 mm, range of PS parameter values equalled 0.0084 mm, minimum error was obtained for the Leitz Reference machine with tactile probe (0.0015 mm) and maximum also for the O-Inspect machine using video probe (0.0099 mm). PS errors obtained for all sensors, except for video probes, had lower values for the Topic White material. Thus, it may be concluded that, for diameter measurements, Topic White should be chosen in order to assure the comparability of optical and tactile coordinate measurements. When using video probes, a slightly better comparability was achieved for the Topic Black material; however, if all three probes considered in this research have to be compared or used together in multisensor measurements, this difference is so low that Topic White should also be chosen in this case.

For measurements of the form deviations of rotary features, the following conclusions were reached. The mean PF error values equalled 0.0010 mm both for the Topic White and Topic Black materials, while for the alumina sphere it equalled 0.0048 mm. Ranges of PF error values were at the level of: 0.0036 mm for the Topic White sphere, 0.0014 mm for the Topic Black sphere and 0.0170 mm for the alumina sphere. In the case of all spheres, the best results regarding the PF parameter value were obtained for O-Inspect with tactile probe head. The worst results were obtained for O-Inspect machine with video probe for the Topic White and Topic Black materials and for the O-Inspect machine equipped with white light sensor in the case of the sphere made of alumina. Basing on that result, it was decided that the best material assuring the comparability of optical and tactile coordinate measurements of the form deviation of rotary features is also the Topic White material. It gives the same mean PF error values as Topic Black but for six out of seven tested combinations of machine and sensor it provides smaller PF error values, oscillating around 0.5 µm, while for Topic Black the PF error values were around 1 µm in most cases.

In the part of research that was related to the determination of sphere centres, the most important parameter showing the level of comparability of optical and tactile coordinate measurements is the difference in centre coordinates obtained for different sensors. In this measurement, the sphere made of the Topic White material also provided the best results. Differences between sphere centre coordinates measured using tactile and video probes were within 3 µm (it was about 4 µm for sphere made of alumina and almost 17 µm for sphere made of topic black) and within 5 µm for sphere centre coordinates measured using tactile and white light sensor (in case of these two sensors similar results were also obtained for spheres made of alumina and topic black). In addition, the standard deviations values of sphere centre coordinates measurement were the lowest for this material.

The last test that was run within the research presented in this paper involved the analysis of the repeatability of the measuring point. From Figure 8, Figure 9 and Figure 10, it may be seen that the repeatability of point measurement is similar for the measurements of the spheres made of alumina and Topic White for all the kinds of considered sensors and it is much bigger for measurements of topic black sphere. Additionally, all measuring points measured using different sensors are the most concentrated in case of the Topic White sphere. All the points measured on this sphere may be located in a square with a side length of 5 µm (for the alumina sphere, the points may be located in a rectangle of side lengths of 13 µm and 7 µm and for the Topic Black sphere in a rectangle of side lengths of 11 µm and 8 µm).

From the practical point of view, the findings of the research presented in this paper are important for people who deal with the accuracy assessment of measurements (including calibration measurements) performed in multisensor mode. In most accuracy assessment methods, relevant material standards have to be used in order to determine the uncertainty of performed measurements (feature-based standards, especially, seem to be a good solution to this problem). Materials selected in this paper in relation to chosen measuring tasks should be the first choice when a decision on the material from which the standard is manufactured has to be made (as a main material or as a coating). This material should also be chosen for the manufacturing of standards that are used for assuring the coincidence of the coordinate systems of different sensors. Especially when tactile, video probe and white light sensors are in use on the considered machine.

As a direction of further works in this subject, it is planned to work on the standards mentioned above and perform complex measurements of them. The level of accuracy improvement achieved thanks to the usage of these standards will be assessed in the scope of future research. The authors plan also to test spheres made of other materials than the ones included in this research. A few promising materials that may be used for manufacturing of standards were found. In the next step, standards will be manufactured using these materials and measurements similar to those presented in this paper will be performed. It is also planned to check the comparability of tactile and optical measurements for measuring tasks other than distance, form and point coordinate measurements.

## Figures and Tables

**Table 1:** Characteristics of the calibration spheres.

Material	Alumina	TOPIC White	TOPIC Black
Deviation of nominal dimension	±0.1 mm	±0.1 mm	±0.1 mm
Measurement uncertainty	0.3 µm	0.3 µm	0.3 µm
Roundness	<0.13 µm	<0.5 µm	<0.2 µm
Roundness uncertainty	0.04 µm	0.04 µm	0.04 µm
Coefficient of thermal expansion	4.6 × 10^−6^ K^−1^	5 × 10^−6^ K^−1^	5 × 10^−6^ K^−1^
Hardness	2100 HV	1000 HV	1800 HV
Recommended measurement method	tactile	optical	optical and tactile

**Table 2:** Sensor types, measuring volumes and specifications of CMMs used in experiment. In E_E0,MPE_ column L is measured distance given in mm.

CMM	Measuring Range, mm	E_E0,MPE_, µm	Sensors	Size MPE, µm	Form MPE, µm
Multisensor Werth VideoCheck HA	600 × 600 × 350	0.5 + *L*/600	Video probe	1.5	1.3
Leitz Reference HP	1000 × 700 × 600	0.7 + *L*/400	Tactile measuring head	0.5	0.5
Multisensor Zeiss O-Inspect 442	400 × 400 × 200	1.9 + *L*/250	Tactile measuring head	1.9	1.9
			Video probe	1.9	1.4
			Chromatic white light sensor	2.0	2.0
Leitz PMM 12106	1200 × 1000 × 600	0.8 + *L*/400	Tactile measuring head	0.8	0.6

**Table 3:** Determination of sphere centres for measurements of spheres made of different materials performed using different sensors.

Standard Sphere	Operation Mode	Parameter	x	y	z
Alumina	Optic	mean, mm	0.0040	0.0016	−0.0001
		standard deviation, mm	0.0018	0.0005	0.0000
	Tactile	mean, mm	0.0000	0.0001	−0.0014
		standard deviation, mm	0.0001	0.0001	0.0028
	WLS	mean, mm	−0.0041	0.0015	0.0030
		standard deviation, mm	0.0008	0.0007	0.0031
Topic white	Optic	mean, mm	0.0028	0.0013	−0.0001
		standard deviation, mm	0.0014	0.0004	0.0000
	Tactile	mean, mm	0.0000	0.0001	−0.0012
		standard deviation, mm	0.0001	0.0000	0.0016
	WLS	mean, mm	−0.0046	0.0018	−0.0006
		standard deviation, mm	0.0001	0.0001	0.0005
Topic black	Optic	mean, mm	0.0064	0.0171	0.0000
		standard deviation, mm	0.0139	0.0092	0.0001
	Tactile	mean, mm	−0.0001	0.0003	−0.0002
		standard deviation, mm	0.0001	0.0001	0.0021
	WLS	mean, mm	−0.0047	0.0014	0.0013
		standard deviation, mm	0.0003	0.0002	0.0006

## Data Availability

Available from corresponding author on request.
